# Neuromuscular symptoms in patients with *RYR1*-related malignant hyperthermia and rhabdomyolysis

**DOI:** 10.1093/braincomms/fcac292

**Published:** 2022-11-10

**Authors:** Luuk R van den Bersselaar, Heinz Jungbluth, Nick Kruijt, Erik-Jan Kamsteeg, Miguel A Fernandez-Garcia, Susan Treves, Sheila Riazi, Ignacio Malagon, Lucas T van Eijk, Nens van Alfen, Baziel G M van Engelen, Gert-Jan Scheffer, Marc M J Snoeck, Nicol C Voermans

**Affiliations:** Malignant Hyperthermia Investigation Unit, Department of Anesthesiology, Canisius Wilhelmina Hospital, 6532 SZ Nijmegen, The Netherlands; Department of Neurology, Donders Institute for Brain, Cognition and Behaviour, Radboud University Medical Center, 6525 GA, Nijmegen, The Netherlands; Department of Paediatric Neurology, Neuromuscular Service, Evelina Children's Hospital, Guy’s and St Thomas’ Hospital NHS Foundation Trust, SE1 7EH London, UK; Randall Centre of Cell and Molecular Biophysics, Muscle Signaling Section, Faculty of Life Sciences and Medicine (FoLSM), King's College, WC2R 2LS London, UK; Department of Neurology, Donders Institute for Brain, Cognition and Behaviour, Radboud University Medical Center, 6525 GA, Nijmegen, The Netherlands; Department of Human Genetics, Radboud University Medical Center, 6525 GA, Nijmegen, The Netherlands; Department of Paediatric Neurology, Neuromuscular Service, Evelina Children's Hospital, Guy’s and St Thomas’ Hospital NHS Foundation Trust, SE1 7EH London, UK; Departments of Biomedicine and Neurology, Neuromuscular research Group, University Hospital Basel, 4031 Basel, Switzerland; Department of Anesthesia, Malignant Hyperthermia Investigation Unit, University Health Network, University of Toronto, M5s 1a4 Toronto, Ontario, Canada; Department of Anesthesiology, Pain and Palliative Medicine, Radboud University Medical Center, 6525 GA, Nijmegen, The Netherlands; Department of Anesthesiology, Pain and Palliative Medicine, Radboud University Medical Center, 6525 GA, Nijmegen, The Netherlands; Department of Neurology, Donders Institute for Brain, Cognition and Behaviour, Radboud University Medical Center, 6525 GA, Nijmegen, The Netherlands; Department of Neurology, Donders Institute for Brain, Cognition and Behaviour, Radboud University Medical Center, 6525 GA, Nijmegen, The Netherlands; Department of Anesthesiology, Pain and Palliative Medicine, Radboud University Medical Center, 6525 GA, Nijmegen, The Netherlands; Malignant Hyperthermia Investigation Unit, Department of Anesthesiology, Canisius Wilhelmina Hospital, 6532 SZ Nijmegen, The Netherlands; Department of Neurology, Donders Institute for Brain, Cognition and Behaviour, Radboud University Medical Center, 6525 GA, Nijmegen, The Netherlands

**Keywords:** exertional rhabdomyolysis, malignant hyperthermia, myopathy, *RYR1*, ryanodine receptor-1

## Abstract

Malignant hyperthermia and exertional rhabdomyolysis have conventionally been considered episodic phenotypes that occur in otherwise healthy individuals in response to an external trigger. However, recent studies have demonstrated a clinical and histopathological continuum between patients with a history of malignant hyperthermia susceptibility and/or exertional rhabdomyolysis and *RYR1*-related congenital myopathies. We hypothesize that patients with a history of *RYR1*-related exertional rhabdomyolysis or malignant hyperthermia susceptibility do have permanent neuromuscular symptoms between malignant hyperthermia or exertional rhabdomyolysis episodes. We performed a prospective cross-sectional observational clinical study of neuromuscular features in patients with a history of *RYR1*-related exertional rhabdomyolysis and/or malignant hyperthermia susceptibility (*n* = 40) compared with healthy controls (*n* = 80). Patients with an *RYR1*-related congenital myopathy, manifesting as muscle weakness preceding other symptoms as well as other (neuromuscular) diseases resulting in muscle weakness were excluded. Study procedures included a standardized history of neuromuscular symptoms, a review of all relevant ancillary diagnostic tests performed up to the point of inclusion and a comprehensive, standardized neuromuscular assessment. Results of the standardized neuromuscular history were compared with healthy controls. Results of the neuromuscular assessment were compared with validated reference values. The proportion of patients suffering from cramps (*P* < 0.001), myalgia (*P* < 0.001) and exertional myalgia (*P* < 0.001) was higher compared with healthy controls. Healthcare professionals were consulted because of apparent neuromuscular symptoms by 17/40 (42.5%) patients and 7/80 (8.8%) healthy controls (*P* < 0.001). Apart from elevated creatine kinase levels in 19/40 (47.5%) patients and mild abnormalities on muscle biopsies identified in 13/16 (81.3%), ancillary investigations were normal in most patients. The Medical Research Council sum score, spirometry and results of functional measurements were also mostly normal. Three of 40 patients (7.5%) suffered from late-onset muscle weakness, most prominent in the proximal lower extremity muscles. Patients with *RYR1* variants resulting in malignant hyperthermia susceptibility and/or exertional rhabdomyolysis frequently report additional neuromuscular symptoms such as myalgia and muscle cramps compared with healthy controls. These symptoms result in frequent consultation of healthcare professionals and sometimes in unnecessary invasive diagnostic procedures. Most patients do have normal strength at a younger age but may develop muscle weakness later in life.

## Teaser

Van den Bersselaar *et al*. report that patients with RYR1-related malignant hyperthermia susceptibility and/or exertional rhabdomyolysis frequently report neuromuscular symptoms such as myalgia and muscle cramps. This results in consultation of healthcare professionals and sometimes in unnecessary invasive diagnostic procedures.

## Graphical Abstract

**Graphical abstract:**
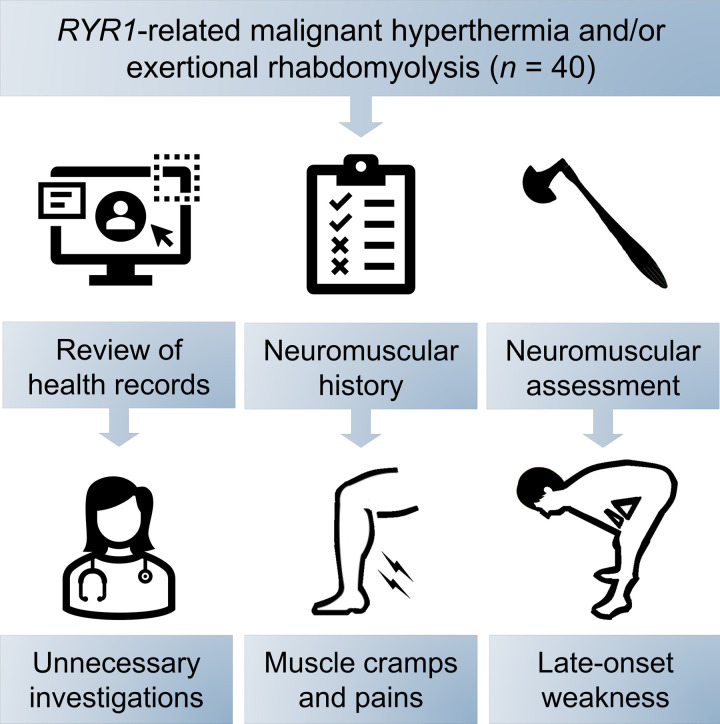

## Introduction

Variants in *RYR1*, the gene encoding the skeletal muscle ryanodine receptor, can give rise to a range of calcium metabolism disturbances in skeletal muscle leading to a wide spectrum of congenital myopathies.^1^ *RYR1* variants also account for a substantial proportion of patients presenting with episodic phenotypes such as exertional rhabdomyolysis^2–4^ (ERM) and malignant hyperthermia (MH).^5,6^ MH is a pharmacogenetic disorder that clinically manifests as a potentially life-threatening hypermetabolic reaction after exposure to volatile anaesthetics or succinylcholine in MH susceptible individuals.^7^ ERM and MH have conventionally been considered episodic phenotypes that occur in otherwise healthy individuals carrying gain-of-function *RYR1* variants in response to an external trigger such as pharmacological agents, strenuous exercise, viral illnesses or a combination of the above.^2,8,9^

However, recent studies have demonstrated a clinical and histopathological continuum between patients with a history of MH susceptibility and/or ERM and *RYR1*-related congenital myopathies.^10,11^ Moreover, in a recent questionnaire study, patients with *RYR1*-related MH susceptibility and/or ERM scored higher on fatigue measures and experienced more social and physical difficulties compared with healthy controls.^12^ In addition to (exertional) myalgia,^2,11,13,14^ these patients may also develop axial muscle weakness later in life.^15^ As previous studies have focused on highly selected patient cohorts or isolated patients presenting with such features, the prevalence and severity of neuromuscular symptoms in an unselected cohort of patients with *RYR1*-related MH susceptibility or ERM is unknown.

Based on previous preliminary observations^2,6,11,12^, we hypothesized that patients with a history of *RYR1*-related MH susceptibility and/or ERM frequently do have neuromuscular symptoms in between MH and/or ERM episodes. This prospective, cross-sectional, observational clinical study aimed to study neuromuscular symptoms and the healthcare burden caused by such symptoms in patients with a history of *RYR1*-related MH susceptibility and/or ERM. We expect our study to provide important insights into the type and frequency of neuromuscular symptoms in patients carrying gain-of-function *RYR1* variants associated with MH susceptibility and/or ERM, and to contribute to optimizing the diagnostic work-up and counselling for such patients.

## Materials and methods

### Standard protocol approvals, registrations and patient consents

This study was approved by the regional medical ethics committee (Research Ethics Board Arnhem–Nijmegen, registration number 2020–6251). All participants provided written informed consent including authorization for the publication of all recognizable persons in photographs and videos according to the Declaration of Helsinki. The study protocol was pre-registered at ClinicalTrials.gov (ID: NCT04610619). This study focusing on the neuromuscular features of *RYR1*-related MH susceptibility and ERM is part of a four-part project aimed at delineating the wider phenotype of *RYR1*-related MH susceptibility and ERM combining questionnaire studies, clinical studies, imaging studies and immunological studies. The study protocol of the parent project has already been published.^16^ Study procedures relevant for this report are reported in Part 1 ‘questionnaire studies’ and Part 2: ‘Clinical studies’. After the publication of the study protocol,^16^ an amendment was made to the study design. The standardized history of neuromuscular symptoms questionnaire (see ‘Study procedures’), was filled out by patients and healthy controls because there are no validated questionnaires available on neuromuscular symptoms.

### Study design and participant recruitment

Patients were recruited and evaluated according to the methods described.^16^ The current study was a prospective, cross-sectional, observational and clinical study. Patients were recruited between August 2020 and June 2021 from the MH cohort seen at the MH Investigation Unit in the Canisius Wilhelmina Hospital, Nijmegen, The Netherlands and from the ERM cohort seen at the neuromuscular clinic of the Radboud University Medical Centre, Nijmegen, The Netherlands. The MH Investigation Unit in the Canisius Wilhelmina Hospital is the only MH Investigation Unit in the Netherlands, and the Human Genetics department at the Radboud University Medical Centre performs *RYR1* sequencing for all Dutch hospitals. These two centres therefore cover all patients with *RYR1* variants in the Netherlands.

For the standardized history of neuromuscular symptoms (see ‘Study procedures’), prospectively matched healthy controls with similar demographic characteristics were recruited among healthy hospital employees from different professional groups.

Inclusion criteria for the patients with a history of *RYR1*-related MH susceptibility and/or ERM were:

- A history of MH susceptibility according to the European Malignant Hyperthermia Group (EMHG) guideline^17^ and/or ERM meeting the RHABDO criteria.^18^ ERM was defined as at least one episode of the following clinical symptoms: cramps, myalgia, myoglobinuria, muscle weakness and/or muscle swelling in combination with an acute creatine kinase (CK) increase (>10 000 U/L) and a subsequent fall of the CK level
- *RYR1* variant(s), identified by whole exome, targeted, partial or full *RYR1* sequencing and classified as pathogenic, likely pathogenic or variant of unknown significance for MH susceptibility^19^
- The ability to speak, read, write and understand Dutch
- Age ≥ 18 years old

Patients were excluded if they fulfilled one or more of the following criteria:

- An initial presentation of an *RYR1*-related congenital myopathy, manifesting as muscle weakness preceding other symptoms
- Other (neuromuscular) diseases resulting in muscle weakness
- A contra-indication for MRI, because of the imaging studies included in the parent study^16^
- Symptoms of angina pectoris
- Current malignancy
- Use of systemic steroids for a period > 14 days during the last 3 months
- Pregnancy or breast-feeding

To prevent inter-family differences or confounding co-morbidities of genetic origin other than *RYR1*-related disease, only one participant per family was included.^20^ If two or more patients from the same family were willing to participate and matched the inclusion criteria, the patient who responded first was included in the study.

The exclusion criteria listed above also applied to healthy controls participating in the standardized history of neuromuscular symptoms part of the study. In addition to these criteria, healthy controls were also excluded if they fulfilled one or more of the following criteria:

- A personal or family history of a confirmed variant in *RYR1*
- A personal or family history of MH susceptibility
- A personal or family history of ERM
- A personal or family history of anaesthetic adverse events suspect to be MH

### Study procedures

**Figure 1:**
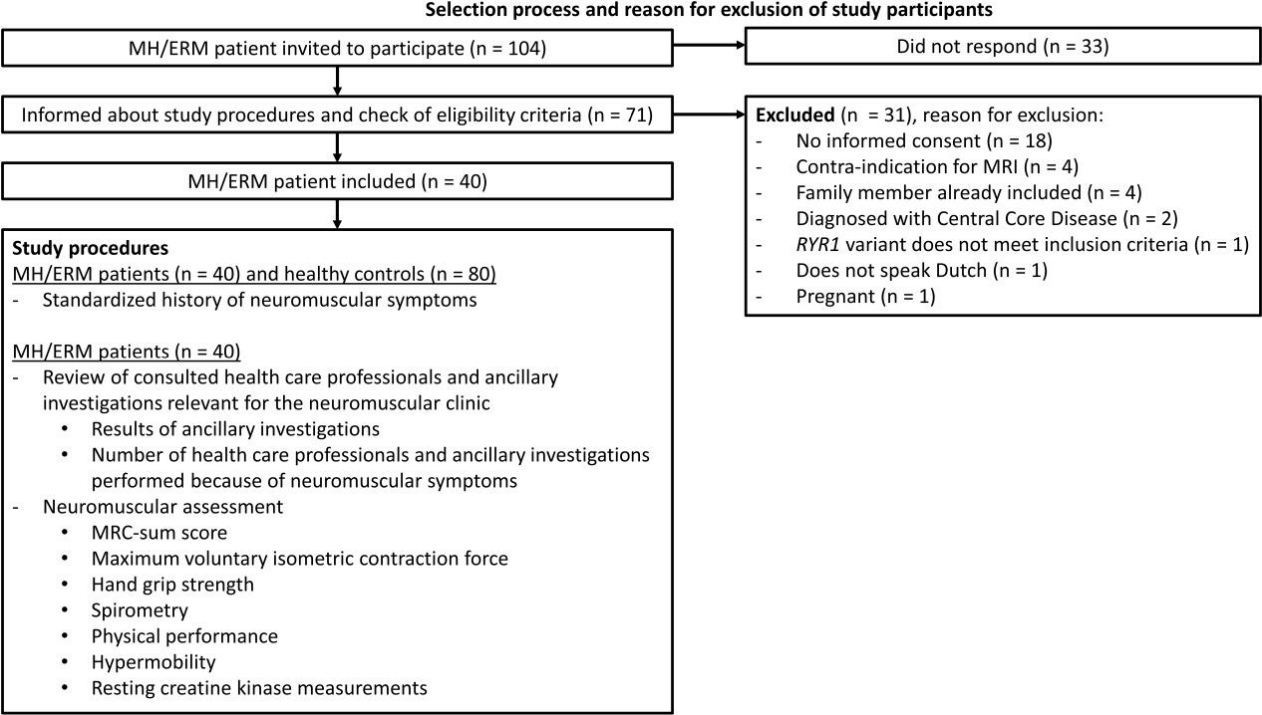
**Selection process and reason for exclusion of study participants.** A summary of the selection process and exclusion criteria for this study. ERM = exertional rhabdomyolysis; MH = malignant hyperthermia; MRI = magnetic resonance imaging.

All participants, patients and healthy controls had a standardized history of neuromuscular symptoms taken. The patients with a history of *RYR1*-relatated MH susceptibility and/or ERM underwent a comprehensive neuromuscular assessment as detailed below. Demographic details (age, sex, country of origin, body weight and height), general information concerning the medical history, current medication and results of all ancillary diagnostic tests relevant for neuromuscular diagnostics were collected from the electronic health records. The study design is summarized in Fig. 1.

### Neuromuscular symptoms

A standardized history of neuromuscular symptoms was taken using a digital questionnaire that all participants filled out at home. The questionnaire contained 11 questions. All questions were closed-ended, with only one mandatory answer per question to complete the questionnaire. The same questionnaire was completed by patients and healthy controls. The full questionnaire, translated from Dutch to English, is available as Supplementary File 1.

### Healthcare professionals consulted, and ancillary diagnostic tests performed before inclusion

Information regarding healthcare professionals consulted, and the results from all ancillary diagnostic tests relevant to neuromuscular diagnostics, were extracted from the patient electronic health records. Subsequently, the number and type of consulted healthcare professionals and diagnostic tests performed for neuromuscular symptoms (e.g. muscle biopsies, electromyography, MRI etc.) up to the point of inclusion were evaluated. Tests performed and physicians consulted for the specific diagnostic work-up to confirm MH susceptibility, or to investigate the aetiology of an ERM episode, were excluded from the latter analysis (e.g. muscle biopsies taken during the *in vitro* contracture test (IVCT) or a neuromuscular assessment after an ERM episode), as they would not reflect the healthcare burden and expenditure because of the primary neuromuscular symptoms we aimed to chart.

### Neuromuscular assessment

The degree and distribution of muscle weakness were documented using the Medical Research Council (MRC)-sum score.^21^ In addition, quantitative measurements of the maximum voluntary isometric contraction force^22^ was performed in the shoulder adductors, elbow flexors and extensors and knee flexors and extensors using a KAP-S sensor (Angewandte System Technik GmbH Gruppe®, Dresden, Germany) connected to a TCIKIT Transducer Interface, DA100C amplifier module and Biopack MP160 system with AcqKnowledge® Software version 5.0.1 (Biopack, Goleta, CA, USA). Hand grip strength was measured using a JAMAR® Plus + Digital Hand Dynamometer (Performance Health, Warrenville, IL, USA). Respiratory muscle strength was tested using spirometry (SpiroUSB, Vyaire Medical connected to PC Spirometry software, Spida CareFusion version 2.3.0.10 for Windows 7). Vital capacity (VC), forced vital capacity (FVC), forced expiratory volume in the first second (FEV1) were measured in the sitting position and in the supine position.^23^ All strength measurements of maximum voluntary isometric contraction,^22^ handgrip strength^24^ and spirometry were performed three times with the highest value used for statistical analysis.

Physical performance was assessed by the timed up and go (TUG) test,^25,26^ the 30 s sit-to-stand test^27^ and the 6-min walking test (6-MWT).^28^ Resting CK levels were measured. Joint hypermobility was rated using the Bulbena^29^ and Beighton score.^30^

### Timing and setting of the study procedures

All study procedures were performed by one physician (LB) during a single visit at the neuromuscular outpatient clinic of the Radboud University Medical Centre, Nijmegen, The Netherlands. Since we aimed to study whether patients with a personal history of *RYR1*-related MH susceptibility and/or ERM suffer from preceding or intermittent neuromuscular symptoms, all study procedures were performed in clinically stable conditions. One patient had an ERM event prior to inclusion. Therefore, the study visit was rescheduled 3 months after the ERM event. The other patients did not have any MH/ERM event in the last year.

### Statistical analysis

The statistical analysis was performed using IBM SPSS (IBM Corp, IBM SPSS Statistics for Windows, version 25.0. Armonk, NY). Demographic characteristics, results of the standardized history of neuromuscular symptoms, the neuromuscular assessment and the electronic health records review were summarized using descriptive statistics. Continuous variables were summarized by their median and interquartile ranges (IQR). Categorical data, including ordinal data, were summarized in terms of numbers and percentages. Demographic characteristics of the patients were compared with healthy controls using the Mann–Whitney U test for continuous variables and the χ^2^ test for categorical data; for this analysis, *P*-values <0.05 were considered significant. Results of the standardized history of neuromuscular symptoms of patients were analysed as follows: for each symptom, the proportion of patients reporting this symptom was compared with healthy controls using the χ^2^ or Fisher’s exact test as appropriate followed by Bonferroni correction for multiple testing. *P*-values < 0.0045 (0.05/11 = 0.0045) were considered significant. Neuromuscular assessment results were compared with validated reference values. For continuous variables, the number and percentage of participants with an abnormal test result [below the lower limit of normal (LLN) or above the upper limit of normal (ULN)], defined as the populations’ 5th and 95th percentiles, are presented using descriptive statistics. Percentage predicted values (% of predicted) were calculated for the MRC-sum score, the TUG test, the 30 s sit-to-stand test, 6-MWT, maximum voluntary isometric contraction force and hand grip strength.

Due to the explorative nature of this study, we did not perform a power calculation. The sample size was based on a previous questionnaire study,^12^ histopathological study^10^ and several case series.^2,11^ These studies with approximately 40 participants showed a substantial clinical and histopathological continuum between patients with *RYR1*-related myopathies and MH susceptibility and/or ERM. Therefore, the sample size was set at 40 patients with a history of MH susceptibility and/or ERM. For the standardized history of neuromuscular symptoms, 80 healthy controls were included. The number of healthy controls was double the number of patients as the variability in the results of healthy controls was expected to be higher in the general population than in patients.

### Data availability

The anonymized dataset generated during this study is available from the corresponding author on reasonable request from qualified investigators.

## Results

### Participants

A total of 104 patients with a history of *RYR1*-related MH susceptibility and/or ERM were invited to participate, of whom 71 responded and were informed about the study procedures. After the informed consent procedure and check of eligibility criteria, 40 patients were included. The patient selection process, reason for exclusion and study design are summarized in Fig. 1.

The proportion of male participants (*P* = 0.693), age (*P* = 0.103) and height (*P* = 0.572) of the patients compared with the healthy controls did not show any statistically significant differences. The weight (*P* = 0.025) and body mass index (BMI) (*P* = 0.001) of the patients were higher compared with healthy controls. In 39 patients (97.5%), MH susceptibility was confirmed by either an EMHG diagnostic *RYR1* variant for MH^31,32^ (*n* = 34; 85%) or a positive IVCT (*n* = 21; 52.5%).^17,33^ Some patients (*n* = 16; 40%), who were investigated with IVCT, were at a later time found to carry diagnostic *RYR1* variants for MH. The only patient in whom MH susceptibility was not confirmed had a history of two ERM episodes and carried the *RYR1* variant c.6710G > A, p.Cys2237Tyr, and is now awaiting IVCT. This *RYR1* variant has been previously associated with MH,^34,35^ and is predicted to have a damaging effect on ryanodine receptor-1 function.^36^ The demographic and genetic characteristics of all study participants are presented in Table 1.

**Table 1:** Demographic and genetic characteristics

	*RYR1*-related MH/ERM (*n* = 40)	Healthy controls (*n* = 80)	*P-*value
**Median age** (years) [IQR^a^]	48 [36–57]	44 [33–52]	0.103
**Male sex**, *n* (%)	25 (62.5)	47 (58.8)	0.693
**Median height** (cm) [IQR^a^]	178 [169–183]	178 [170–185]	0.572
**Median weight** (kg) [IQR^a^]	87 [73–100]	77 [67–86]	0.025
**Median body mass index** (kg/m^2^) [IQR^a^]	28 [24–31]	24 [23–27]	0.001
**Country of origin**, *n* (%)			
Netherlands, *n* (%)	36 (90.0)	73 (91.3)	-
Afghanistan, *n* (%)	0 (0)	1 (1.3)	–
Aruba, *n* (%)	0 (0)	1 (1.3)	–
Belgium, *n* (%)	0 (0)	1 (1.3)	-
Indonesia, *n* (%)	1 (2.5)	0 (0)	–
Syria, *n* (%)	0 (0)	1 (1.3)	–
Turkey, *n* (%)	1 (2.5)	0 (0)	–
Netherlands—Austria, *n* (%)	0 (0)	1 (1.3)	–
Netherlands—Germany, *n* (%)	0 (0)	1 (1.3)	–
Netherlands—Indonesia, *n* (%)	1 (2.5)	1 (1.3)	–
Netherlands—Curacao, *n* (%)	1 (2.5)	0 (0)	–
**Malignant hyperthermia susceptibility confirmed**, *n* (%)	39 (97.5)	–	–
**Personal history of malignant hyperthermia reaction**, *n* (%)	9 (22.5)	–	–
**Personal history of exertional rhabdomyolysis event**, *n* (%)	6 (15.0)	–	–
**EMHG diagnostic *RYR1* variants for malignant hyperthermia**, *n* (%)	34 (85)	–	–
c.14545G > A, p.Val4849Ile, *n* (%)	16 (40.0)	–	–
c.1021G > A, p.Gly341Arg, *n* (%)	5 (12.5)	–	–
c.6617C > T, p.Thr2206Met, *n* (%)	4 (10.0)	–	–
c.38T > G, p.Leu13Arg, *n* (%)	3 (7.5)	–	–
c.1840C > T, p.Arg614Cys, *n* (%)	2 (5.0)	–	–
c.7300G > A, p.Gly2434Arg, *n* (%)	2 (5.0)	–	–
c.7361G > A, p.Arg2454His, *n* (%)	1 (2.5)	–	–
c.38T > G, p.Leu13Arg and c.6419G > A, p.Arg2140Gln (Trans), *n* (%)	1 (2.5)	–	–
**Other *RYR1* variants**, *n* (%)	6 (15)	–	–
c.14210G > A, p.Arg4737Gln and c.4178A > G, p.Lys1393Arg (Cis), *n* (%)	2 (5.0)	–	–
c.1024G > A, p.Glu342Lys, *n* (%)	1 (2.5)	–	–
c.12226C > T, p.Phe4076Leu, *n* (%)	1 (2.5)	–	–
c.10616G > A, p.Arg3539His, *n* (%)	1 (2.5)	–	–
c.6710G > A, p.Cys2237Tyr, *n* (%)	1 (2.5)	–	–

Summary of the age, sex, height, body weight, body mass index and country of origin of all study participants. The number of malignant hyperthermia and exertional rhabdomyolysis events and *RYR1* variants identified in the patients with a history of *RYR1*-related malignant hyperthermia susceptibility and/or exertional rhabdomyolysis are also listed. The *RYR1* variants are categorized as diagnostic for malignant hyperthermia according to the European Malignant Hyperthermia Group list of diagnostic *RYR1* variants and other variants. ^a^IQR = interquartile range. ^b^EMHG = European Malignant Hyperthermia Group.

### Neuromuscular symptoms

**Figure 2:**
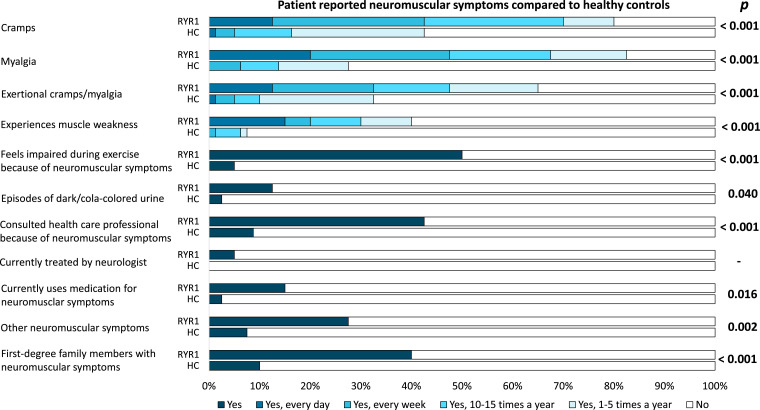
**Patient reported neuromuscular symptoms compared with healthy controls.** Neuromuscular symptoms as reported in a standardized history (*n* = 40). For each symptom, the proportion of patients reporting this symptom was compared with healthy controls (*n* = 80) using the χ^2^ or Fisher’s exact test, when appropriate followed by Bonferroni correction for multiple testing (*P*-values < 0.0045 were considered significant). The full questionnaire translated from Dutch to English is available as Supplementary File 1.

**Figure 3:**
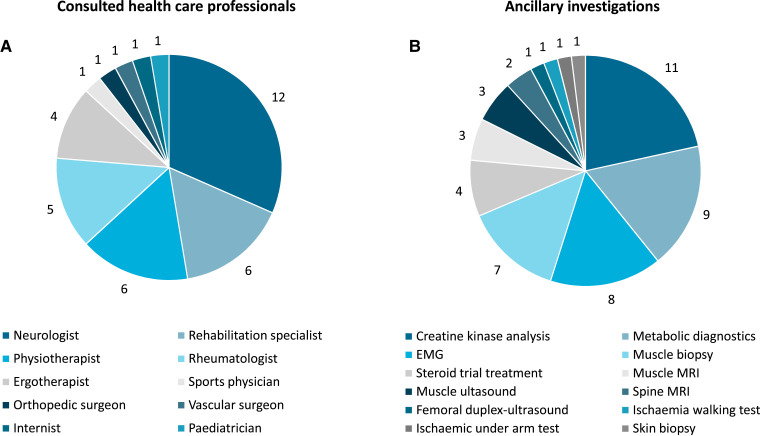
**Healthcare professionals consulted, and ancillary investigations performed because of neuromuscular symptoms before inclusion in this study.** A summary of all healthcare professionals consulted (**A**) and ancillary investigations performed (**B**) in the study participants. All healthcare professionals were consulted, and ancillary investigations were performed because of neuromuscular symptoms before inclusion. Investigations and physicians during the diagnostic work-up to diagnose malignant hyperthermia susceptibility or to investigate the aetiology of an exertional rhabdomyolysis episode were excluded.

The proportion of patients suffering from cramps (*P* < 0.001), myalgia (*P* < 0.001) and exertional myalgia (*P* < 0.001) was higher compared with healthy controls. Furthermore, the proportion of patients that experienced muscle weakness (*P* < 0.001) and felt impaired during exercise (*P* < 0.001) was higher compared with healthy controls. Episodes of dark/cola-coloured urine were reported in a similar proportion of patients (five of 40; 12.5%) and healthy controls (two of 80; 2.5%) (*P* = 0.040). Healthcare professionals were consulted because of neuromuscular symptoms by 17 of 40 (42.5%) patients and seven of 80 (8.8%) healthy controls (*P* < 0.001). The details of the results of the standardized history of neuromuscular symptoms are summarized in Fig. 2. The patients consulted a wide spectrum of healthcare professionals as summarized in Fig. 3A and discussed below. The healthy controls consulted a physiotherapist (*n* = 4) or a general practitioner (*n* = 3) for symptoms of myalgia and cramps. Current use of medications (e.g. analgesics, clonazepam, dantrolene or magnesium supplements) to treat neuromuscular symptoms was reported by 6 of 40 (15%) patients and two of 78 (2.6%) healthy controls, this was not statistically significant (*P* = 0.016) after adjustment for multiple testing. The two healthy control subjects both used magnesium supplements to treat cramps. Neuromuscular symptoms in first-degree family members were more frequently reported by patients (16 of 40; 40%) compared with healthy controls (eight of 80; 10%) (*P* < 0.001).

Subgroup analysis of the standardized history of neuromuscular symptoms of patients with a personal history of an ERM or MH episode (*n* = 15) compared with patients with MH susceptibility but without a personal history of any ERM or MH episodes (*n* = 25) did not show any significant differences.

### Ancillary investigations performed and healthcare professionals consulted before inclusion

Apart from mild abnormalities identified in 13 of 16 (81.3%) muscle biopsies taken from 15 patients the results of ancillary investigations relevant for neuromuscular diagnostics were normal in most patients. Abnormal test results were not only identified in patients with but also in those without regular cramps and myalgia. The results of ancillary investigations relevant for neuromuscular diagnostics, the number of patients with cramps and/or myalgia on a daily or weekly basis and the *RYR1* variants identified in these patients are summarized in Table 2.

**Table 2:** Results of all ancillary investigations, neuromuscular symptoms and *RYR1* variants identified

	Number of patients with cramp/myalgia on a daily/weekly basis	*RYR1* variants identified in the patients with cramp/myalgia on a daily/weekly basis
Muscle biopsy results available (*n* = 16)		
Normal (*n* = 3)	2/3	p.Gly2434Arg (2x)
Biopsies with myopathic features^a^ (*n* = 13); details available in Supplementary Table 1	6/13	Details available in Supplementary Table 1
Metabolic diagnostics relevant for the neuromuscular diagnostics (*n* = 14)		
Comprehensive screening package for metabolic disorders (*n* = 6)		
Normal (*n* = 5)	1/5	p.Val4849Ile
Elevated circulating alanine and carnitine (*n* = 1)	1/1	p.Val4849Ile
Thyroid stimulating hormone measurements (*n* = 5)		
Normal (*n* = 4)	1/4	p.Val4849Ile
Elevated (*n* = 1)	1/1	p.Val4849Ile
Myositis line blot immunoassay (*n* = 3)		
Normal (*n* = 3)	3/3	p.Cys2237Tyr; p.Gly2434Arg; p.Phe4076Leu
Electromyography results available (*n* = 8)		
Normal (*n* = 7)	3/7	p.Gly2434Arg; p.Phe4076Leu; p.Val4849Ile
Minor spontaneous activity (*n* = 1)	0/1	NA
Muscle ultrasound results available (*n* = 3)		
Normal (*n* = 1)	0/1	NA
Hypertrophy, echogenicity normal (*n* = 1)	0/1	NA
Bilateral increased echo intensity of the tibialis anterior and gastrocnemius muscles (*n* = 1)	1/1	p.Gly2434Arg
Muscle MRI results available (*n* = 3)		
Normal (*n* = 2)	1/2	p.Phe4076Leu
Oedema, fatty infiltration, atrophy of the muscles of the lower extremities (*n* = 1)	1/1	p.Gly2434Arg
Spine MRI results available (*n* = 2)		
Normal (*n* = 2)	0/2	NA
Ischemic underarm test results available (*n* = 1)		
Normal (*n* = 1)	0/1	NA
Skin biopsy results available (*n* = 1)		
Normal (*n* = 1)	0/1	NA
Ischemia walking test results available (*n* = 1)		
Normal (*n* = 1)	1/1	p.Gly341Arg
Femoral artery ultrasound-duplex results available (*n* = 1)		
Normal (*n* = 1)	1/1	p.Gly341Arg

The results of relevant ancillary investigations and the number of patients with cramps/myalgia on a daily/weekly basis for each result. Furthermore, the *RYR1* variants identified in these patients are given. These investigations had been performed before the inclusion in this study because of neuromuscular symptoms or the diagnostic work-up for malignant hyperthermia susceptibility or to investigate the aetiology of an exertional rhabdomyolysis episode. Details of the biopsies with myopathic features are available as Supplementary Table 1. ^a^One of the patients had two muscle biopsies from the vastus lateralis muscle left and right with an interval of 4 years, the first (right vastus lateralis muscle) had type 1 fibre type predominance, the second (left vastus lateralis muscle) had minimal non-specific myopathic changes.

A wide spectrum of healthcare professionals had been consulted because of cramps, myalgia or other neuromuscular symptoms in 17 of 40 (42.5%) patients. The three most frequently consulted healthcare professionals were neurologists (*n* = 12), rehabilitation specialists (*n* = 6) and physiotherapists (*n* = 6). A summary of all consulted healthcare professionals is illustrated in Fig. 3A. As shown in this figure, some patients consulted less obvious types of healthcare professionals for diagnosing and treating neuromuscular symptoms. For instance, one patient consulted an orthopaedic surgeon because of calf cramps, a shortened Achilles tendon was considered as the cause and surgical extension was considered. After a comprehensive assessment, surgery was cancelled because her symptoms were considered non-specific. Another patient consulted a vascular surgeon who considered peripheral artery disease to be the cause of his myalgia and cramps. He had a comprehensive assessment including an ischaemia walking test and femoral artery ultrasound-duplex, both of which were normal.

Ancillary tests to diagnose the aetiology of these neuromuscular symptoms were performed in 13 of 40 patients (32.5%). These tests and interventions (summarized in Fig. 3B) ranged from imaging studies (e.g. muscle ultrasound, muscle MRI) to more invasive procedures such as a muscle biopsy or even a steroid trial under the suspicion of an inflammatory myopathy.

### Neuromuscular assessment during the study visit

CK was elevated in 19 of 40 (47.5%) patients with a median resting CK level of 232 IU/L [IQR 167–390]. The MRC-sum score and results of functional measurements (TUG test, 6-MWT, 30-s sit-to-stand test, Bulbena and Beigthon score) were normal in most patients. The handgrip strength was below the reference value in one of 40 (2.5%) patients and above the reference value in 11 of 40 (27.5%) patients. The maximum voluntary isometric contraction force of the elbow flexors was below the ULN in 15 of 40 (37.5%) patients. The results of quantitative measurements of maximum voluntary isometric contraction force of other muscle groups were normal in most patients. During the neuromuscular assessment, 13 of 40 (32.5%) patients suffered muscle cramps triggered by maximum voluntary contraction and/or the hypermobility assessment. The median FVC was 87.6% [IQR 78.8–95.0] of predicted. The median decrease in VC in the supine position was 3.9% [IQR 0.01–8.4]. Results of the neuromuscular assessment are summarized in Table 3.

**Table 3:** Neuromuscular assessment of the participants compared with reference values

	Median [IQR^a^]	Reference value	Participants < LLN^b^, *n* (%)	Participants > ULN^c^, *n* (%)	Median % of predicted [IQR^a^]
Neuromuscular tests					
MRC^d^-sum score	60 [60–60]	60^21^	6 (15)	NA^e^	100.0 [100.0–100.0]
Resting CK^f^ value (iU/L)	232 [167–390]	Pers^d^ ^37^	NA^e^	19 (47.5)	NA^e^
Timed up-and-go (s)	7.4 [6.8–8.2]	Pers^d^ ^25,26^	NA^e^	4 (10)	84.2 [71.6–95.3]
30 s sit-to-stand (freq)	12 [9–13]	Pers^d^ ^27^	11 (27.5)	NA^e^	87.1 [67–102.3]
Six-minute walking test (m)	569 [506–617]	Pers^d^ ^28^	5 (12.5)	NA^e^	93.8 [80.8–106.2]
Handgrip strength (kg)	47.2 [33.6–52.4]	Pers^d^ ^24^	1 (2.5)	11 (27.5)	114.7 [103.6–137.4]
Beighton score	0 [0–0]	M < 4; F < 5^29^	NA^e^	2 (5)	NA^e^
Bulbena score	0 [0–2]	< 5^30^	NA^e^	2 (5)	NA^e^
Maximum voluntary isometric contraction (kg)					
Shoulder adduction right	23.9 [16.2–31.5]	Pers^d^ ^22^	5 (87.5)	NA^e^	99.3 [66.2–117.5]
Shoulder adduction left	25.6 [15.3–30.8]	Pers^d^ ^22^	6 (85)	NA^e^	102.3 [68.8–139.9]
Elbow flexion right	17.5 [14.8–22.4]	Pers^d^ ^22^	14 (35)	NA^e^	77.9 [57.5–94.4]
Elbow flexion left	18.3 [13.7–23.3]	Pers^d^ ^22^	15 (37.5)	NA^e^	79.9 [60.7–99.9]
Elbow extension right	17.5 [13.4–21.8]	Pers^d^ ^22^	3 (7.5)	NA^e^	116.6 [96.7–133.8]
Elbow extension left	17.8 [13.0–22.1]	Pers^d^ ^22^	2 (5)	NA^e^	123.1 [100.6–141.5]
Knee flexion right	18.8 [13.8–23.6]	Pers^d^ ^22^	3 (7.5)	NA^e^	99.8 [80.8–119.2]
Knee flexion left	18.9 [14.8–22.8]	Pers^d^ ^22^	3 (7.5)	NA^e^	108 [80.4–124.4)
Knee extension right	32.9 [25.1–39.3]	Pers^d^ ^22^	9 (22.5)	NA^e^	91.1 [67.6–110.9]
Knee extension left	33.4 [24.2–41.9]	Pers^d^ ^22^	7 (17.5)	NA^e^	97.2 [66.5–122.4]
Spirometry					
FVC^g^ (L)	4.12 [3.51–4.78]	Pers^d23^	NA^e^	NA^e^	87.6 [78.8–95.0]
VC^h^ (L)	4.48 [3.72–5.03]	NA^e^	NA^e^	NA^e^	NA^e^
VC^h^ (L) (% decrease in supine position)	3.9 [0.01–8.4]	NA^e^	NA^e^	NA^e^	NA^e^
FEV^i^ (L)	3.60 [3.00–4.03]	NA^e^	NA^e^	NA^e^	NA^e^
FEV1/FVC	0.88 [0.82–0.93]	NA^e^	NA^e^	NA^e^	NA^e^

Results of the neuromuscular assessment performed during the study visit. Results were compared with normative values as previously reported. Results were considered under the lower limit of normal when under the 5th percentile and were considered above the upper limit of normal when above the 95th percentile. ^a^IQR = interquartile range. ^b^LLN = lower limit of normal; ^c^ULN = upper limit of normal. ^d^Reference values were personalized and corrected for sex, age, weight or length. ^e^NA = not appliable; ^f^CK = creatine kinase; ^g^FVC = forced vital capacity; ^h^VC = vital capacity; ^i^FEV1 = forced expiratory volume during the first second.

Three of 40 (7.5%) patients suffered from severe cramps and myalgia combined with late-onset muscle weakness, most prominent in the proximal lower extremity muscles; those patients were all male and over the age of 45 years. Although all three patients never had a neuromuscular assessment before the onset of muscle weakness, they did only have mild symptoms of myalgia and normal neuromuscular strength at a younger age. In one of these patients who carried the *RYR1* c.12226C > T, p.Phe4076Leu variant, the first muscle biopsy showed type I fibre predominance. The second muscle biopsy, performed 4 years later because of progression of weakness, cramps and myalgia, showed small type I fibres and other minor non-specific changes. The myositis line blot, muscle MRI and EMG were all normal. His relatives were not able and/or willing to undergo *RYR1* sequencing.

**Figure 4:**
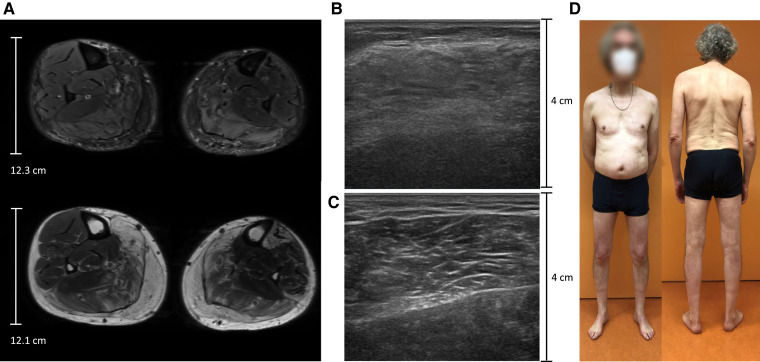
**Magnetic resonance imaging, muscle ultrasound images and clinical photographs from study participants. (A**) Magnetic resonance imaging Dixon T2 images and Dixon T1 images showing bilateral edema of the gastrocnemius and soleus muscle as well as fatty infiltration of the left gastrocnemius, tibialis anterior and semimembranosus muscles. The muscle magnetic resonance imaging and muscle ultrasound (**B and C**) were performed because of late-onset proximal muscle weakness. This patients carries the *RYR1* c.7300G > A, p.Gly2434Arg variant. (**B**) Muscle ultrasound imaging showing markedly increased echo intensity (Heckmatt rating scale^39^ 4) of the right medial gastrocnemius muscle This ultrasound image is from the same patient whose magnetic resonance images are shown in Fig. 4A and whose muscle ultrasound images are shown in Fig. 4C. (**C**) Muscle ultrasound images of the right lateral gastrocnemius muscle showing increased echo intensity (Heckmatt rating scale^39^ 2). This ultrasound image is from the same patient whose magnetic resonance images are shown in Fig. 4A and whose muscle ultrasound images are shown in Fig. 4B. (**D**) A study participant with late-onset proximal weakness and atrophy of the lower extremities as demonstrated in Videos 1 and 2. He carries the *RYR1* c.10616G > A, p.Arg3539His variant.

The second patient who had substantial proximal muscle weakness carried the EMHG diagnostic *RYR1* c.7300G > A, p.Gly2434Arg variant.^38^ He had bilateral edema of the gastrocnemius and the soleus muscles, as well as fatty infiltration of the left gastrocnemius, tibialis anterior and semimembranosus muscles on T1 and T2 weighted Dixon MRI (Fig. 4A). A muscle ultrasound performed in this patient showed bilateral increased echo intensity within the tibialis anterior [Heckmatt rating scale^39^ (HRS) 4 on the right side and HRS 3 on the left side] and gastrocnemius muscles (bilateral lateral gastrocnemius muscles HRS 2, right medial gastrocnemius HRS 4 and left medial gastrocnemius HRS 3) as shown in Fig. 4B and C. Muscle biopsy, myositis line blot and EMG were normal in this patient. His daughter also suffers from myalgia and cramps and his son had delayed motor milestones as a child without further concerns at an older age. Two other children do not have any neuromuscular symptoms. All children are currently awaiting *RYR1* sequencing.

A third patient who carried the *RYR1* c.10616G > A, p.Arg3539His variant also had substantial proximal muscle weakness as well as atrophy of the lower extremities (Fig. 4D). He had never consulted a neurologist or other healthcare professionals. Since approximately 3 years he had experienced problems climbing stairs (Video 1) and was not able to stand up kneeling on one knee (Video 2) or from a chair or without using his arms.

## Discussion

Our findings illustrate that a significant proportion of patients with MH associated *RYR1* gain-of-function variants suffer from frequent neuromuscular symptoms other than those resulting from exposure to MH triggering agents. Whilst fixed myopathic manifestations such as muscle weakness are relatively rare and mainly limited to the older age group, episodic manifestations including (exertional) cramps and myalgia are common. As our study shows these symptoms frequently lead to the consultation of healthcare professionals and (often invasive) diagnostic procedures carried out in addition to those specifically required to confirm MH susceptibility.

Other noteworthy results included abnormal resting CK levels, which were elevated in 19 of 40 (47.5%) patients, and the mild histopathological abnormalities identified in 13 of 16 (81.3%) muscle biopsies taken from 15 patients. Results of other assessments such as spirometry, neuromuscular examinations and the results of previously performed ancillary investigations, were normal in most patients. These findings are in line with previous studies performed in selected cohorts of carriers of *RYR1* gain-of-function variants presenting with symptoms of myalgia and cramps.^2,6,11,13,14^ Interestingly, the myopathic manifestations in the three patients with severe proximal muscle weakness in our study are similar to the neuromuscular symptoms reported recently in a case report of a 60-year-old male with late-onset quadriceps weakness and a history of *RYR1*-related MH.^40^

In a recently performed explorative questionnaire study by our team,^12^ patients with *RYR1*-relelated MH susceptibility or ERM scored higher on fatigue and experienced more social and physical difficulties compared with healthy controls. We did not study the reason for this observation but hypothesized that MH susceptibility and/or ERM are not purely episodic phenotypes but do exhibit myopathic features in between MH and/or ERM episodes, mainly manifesting as myalgia, cramps and/or fatigue but occasionally also as muscle weakness.^12^ The results of our current study in an unselected cohort confirm these preliminary observations.

The high proportion of patients suffering from (exertional) myalgia and cramps within our study cohort might be related to the specific pathophysiological mechanisms underlying *RYR1*-associated MH and ERM, which are both characterized by an uncontrolled calcium release from the sarcoplasmic reticulum in response to external triggers.^3,7^ Several laboratory studies have shown that skeletal muscle cells from MH susceptible patients have higher resting cytosolic calcium concentrations.^41,42^ In one of these studies, an increased spontaneous calcium release from the sarcoplasmic reticulum in myotubes from MH susceptible patients was observed compared with healthy controls. The alterations in calcium metabolism had a moderate correlation (R2 = 0.49) with neuromuscular symptoms^42^ and may contribute to the neuromuscular symptoms as reported in our study. However, future studies are necessary to confirm this.

Laboratory studies on the effects of aging and altered calcium metabolism on late-onset muscle weakness in mice have shown that the higher resting cytosolic calcium concentrations, as observed in skeletal muscle cells of MH susceptible individuals,^41,42^ may cause myofibrillar disarray, sarcolemmal rupture, structural alterations such as cores and, mitochondrial damage.^43,44^ Interestingly, the *RYR1* knock-in mouse model used in one of these studies (p.Gly2435Arg) corresponds with the human *RYR1* c.7300G > A, p.Gly2434Arg variant,^44^ which was also found in one of the patients with late-onset proximal muscle weakness identified in our study. Furthermore, structural alterations in skeletal muscle cells of MH susceptible mice appear to increase in severity with age.^45^ It seems plausible that these effects may also contribute to the late-onset muscle weakness observed in some patients included in our study cohort.

Our observations have a number of important implications for the diagnosis and management of individuals carrying MH-associated *RYR1* variants. Firstly, anaesthesiologists and other professionals looking after MH-susceptible individuals ought to be aware that episodic and permanent neuromuscular manifestations are part of the MH disease spectrum and do not necessarily reflect a second pathology. Patients should be informed about these neuromuscular manifestations with the aim to reduce unnecessary consultations with other healthcare professionals and additional diagnostic investigations. Secondly, gain-of-function *RYR1* variants might be an underrecognized cause of cramps and myalgia. Neurologists, rheumatologists and other professionals to whom such patients may present ought to suspect MH-associated *RYR1* variants in individuals presenting with cramps, myalgia, hyperCKemia, ERM and a positive family history in whom other more obvious causes have been excluded,^6,18,37,46^ thus preventing avoidable and potentially fatal anaesthesia complications.

Our study has a few limitations: In particular, patients with neuromuscular symptoms are probably more motivated to participate in clinical studies resulting in a selection bias. Furthermore, patients were aware of the diagnosis of MH susceptibility and/or have suffered from an ERM event before completing our neuromuscular symptom questionnaire, introducing the possibility of recall bias. In addition, patient demographic characteristics differed from the healthy controls as weight (*P* = 0.025) and therefore BMI (*P* < 0.001) was higher in the patients compared with healthy controls. However, it is unlikely this has affected our results as we are not aware of any association between BMI and neuromuscular symptoms such as cramps and myalgia. This observation could also reflect an increased muscle mass as previously reported in patients with gain-of-function *RYR1* variants.^2^ In addition, hospital employees are generally healthier compared with the general population. Moreover, the healthy controls included in our study did not undergo *RYR1* sequencing, and it is therefore theoretically conceivable that those carried currently unidentified *RYR1* variants. However, given the relatively low prevalence of *RYR1* gain-of-function variants,^47^ this is unlikely to have had a major effect on our results. Finally, we have only included patients with MH susceptibility and/or ERM due to *RYR1* gain-of-function variants, and can therefore not provide any information concerning neuromuscular symptoms in patients with MH susceptibility and/or ERM with variants in other relevant genes such as *CACNA1S*,^48^ or in patients without any relevant genetic variants identified.^4,5,6^ There are currently no case reports or studies indicating similar neuromuscular symptoms in these patients, however, only 2–5% of MH susceptible patients carry *CACNA1S* variants^5,6^ with only few reports of *CACNA1S-*associated rhabdomyolysis and otherwise asymptomatic hyperCKemia.^49,50^

Future studies should focus on the impact of neuromuscular symptoms in patients with *RYR1*-related MH susceptibility and/or ERM on daily life and on the further investigation of underlying pathophysiological mechanism, in particular with regards to the striking phenotypical variability of patients with MH and/or ERM associated *RYR1* variants. Another important aspect concerns the repurposing of already existing drugs and the development of novel and more effective treatments for patients with *RYR1*-related cramps and myalgia. Dantrolene, a ryanodine receptor antagonist and the first line treatment for MH^51^ has been used successfully in some MH susceptible patients with severe cramps and myalgia and might be useful in this respect.^3,52^ Lastly, the emergence of permanent myopathies at an older age suggests a potential synergism between RyR1 dysfunction and the physiological aging process, emphasizing the importance of further longitudinal studies in this cohort extending into older age groups.

## Conclusion

Patients with *RYR1* variants resulting in MH susceptibility and/or ERM frequently report neuromuscular symptoms such as myalgia and muscle cramps. This results in consultation of healthcare professionals and sometimes in unnecessary invasive diagnostic procedures. Most patients do have normal strength at a younger age but may develop muscle weakness later in life.

## Supplementary Material

fcac292_Supplementary_Data

## Abbreviations

6-MWT =: six-minute walking test
BMI =: body mass index
CK =: creatine kinase
EMHG =: European Malignant Hyperthermia Group
EMG =: ERM = exertional rhabdomyolysis
FEV1 =: forced expiratory volume in the first second
FVC =: forced vital capacity
HRS =: Heckmatt rating scale
IQR =: interquartile ranges
IVCT =: *in vitro* contracture test
LLN =: lower limit of normal
MH =: malignant hyperthermia
MRC =: Medical Research Council
TUG =: timed up and go
ULN =: upper limit of normal
VC =: vital capacity
