# Supplementary material for: Neuromuscular symptoms in patients with RYR1-related malignant hyperthermia and rhabdomyolysis
Source: Brain Commun. 2022 Nov 10;4(6):fcac292. doi: 10.1093/braincomms/fcac292 (PMC9897183; doi:10.1093/braincomms/fcac292)
Supplement: fcac292_Supplementary_Data [file fcac292_Supplementary_Data.zip › Supplementary File 1 - Questionnaire.docx]

**Supplementary 1 - Questionnaire for the standardized history on neuromuscular symptoms**

1. Did you have any complaints of muscle cramps during the last year?
   - Yes
   - No

If yes, how frequently do have symptoms of muscle cramps?

- 1 – 5 times a year
- 10 – 15 times a year
- Every week
- Every day

1. Did you have any complaints of myalgia during the last year?
   - Yes
   - No

If yes, how frequently do have symptoms of myalgia?

- 1 – 5 times a year
- 10 – 15 times a year
- Every week
- Every day

1. Did you have any complaints of myalgia or muscle cramps during exercise the last year?
   - Yes
   - No

If yes, how frequently do have symptoms of myalgia?

- 1 – 5 times a year
- 10 – 15 times a year
- Every week
- Every day

1. Have you ever had any episodes of very dark colored urine? (With dark urine we mean the same color as coke).
   - Yes
   - No
2. Did you have any complaints of muscle weakness the last year?
   - Yes
   - No

If yes, how frequently do have symptoms of myalgia?

- 1 – 5 times a year
- 10 – 15 times a year
- Every week
- Every day

1. Did you have any other muscle complaints during the last year?
   - Yes
   - No

If yes, which complaints?

1. Do you feel impaired during exercise because of neuromuscular symptoms?
   - Yes
   - No

If yes, which symptoms?

1. Do you currently use any medication because of neuromuscular symptoms?
   - Yes
   - No

If yes, which symptoms?

1. Are you currently being treated by a neurologist?
   - Yes
   - No
2. Do you have any first-degree family members (children, parents, sisters or brothers) with neuromuscular complaints?
   - Yes
   - No
3. Have you ever consulted a health care professional because of neuromuscular symptoms?
   - Yes
   - No

If yes, which health care professionals?
