# Supplementary material for: Neuromuscular symptoms in patients with RYR1-related malignant hyperthermia and rhabdomyolysis
Source: Brain Commun. 2022 Nov 10;4(6):fcac292. doi: 10.1093/braincomms/fcac292 (PMC9897183; doi:10.1093/braincomms/fcac292)
Supplement: fcac292_Supplementary_Data [file fcac292_Supplementary_Data.zip › Supplementary Table 1 - Biopsies with myopathic features.docx]

| **Supplementary Table 1: Details of biopsies with myopathic features (n = 13)** | | | |
| --- | --- | --- | --- |
|  | | **Number of patients with cramp/myalgia**  **on a daily/weekly basis** | ***RYR1* variants identified in patients with cramp/myalgia on a daily/weekly basis** |
| **Myopathic features** |  | |  |
| Increased number of fibers with internal nuclei (n = 3) | | 1/3 | c.6710G>A, p.Cys2237Tyr |
| Type 1 fiber predominance and increased number of fibers with internal nuclei (n = 2) | | 0/2 | NA |
| Minor, nonspecific myopathic changes (n = 2)^a^ | | 1/2 | c.12226C>T, p.Phe4076Leu |
| Type 1 fiber predominance (n = 1)^a^ | | 1/1 | c.12226C>T, p.Phe4076Leu |
| Lipid droplets (n = 1) | | 0/1 | NA |
| Nemaline rods (n = 1) | | 1/1 | c.14545G>A, p.Val4849Ile |
| Increased number of fibers with internal nuclei and cores (n = 1) | | 0/1 | NA |
| Increased fiber size variability (n = 1) | | 1/1 | c.7361G>A, p.Arg2454His |
| Increased number of fibers with internal nuclei and fiber type disproportion (n = 1) | | 1/1 | c.6617C>T, p.Thr2206Met |

^a^One of the patients had two muscle biopsies from the vastus lateralis muscle left and right with an interval of four years, the first (right vastus lateralis muscle) had type 1 fiber type predominance, the second (left vastus lateralis muscle) had minimal non-specific myopathic changes.
